# Diagnostic Challenges and Insights Into Granulomatous Mastitis: A Systematic Review

**DOI:** 10.7759/cureus.75733

**Published:** 2024-12-15

**Authors:** Nandan M Shanbhag, Mouza A Ameri, Sneha N Shanbhag, Nevine Anandan, Khalid Balaraj, Abdulrahman Bin Sumaida

**Affiliations:** 1 Internal Medicine, College of Medicine and Health Sciences, United Arab Emirates University, Al Ain, ARE; 2 Radiation Oncology/Palliative Care, Tawam Hospital, Al Ain, ARE; 3 Surgery, Tawam Hospital, Al Ain, ARE; 4 Internal Medicine, Dubai Physiotherapy and Rehabilitation Centre, Dubai, ARE; 5 Radiology, Tawam Hospital, Al Ain, ARE; 6 Oncology/Radiation Oncolgy, Tawam Hospital, Al Ain, ARE

**Keywords:** breast malignancy, diagnosis, granulomatous mastitis, idiopathic granulomatous mastitis, inflammation

## Abstract

Granulomatous mastitis (GM) is a chronic inflammatory breast condition that presents significant diagnostic challenges due to its clinical and imaging similarities to malignancies. Accurate diagnosis is crucial to avoid unnecessary interventions and ensure effective management. A total of 1,216 articles were initially identified through a comprehensive database search. After removing duplicates and conducting a systematic review, 73 studies were shortlisted for full-text evaluation, with 31 studies meeting the inclusion criteria. These studies were analyzed to extract data on diagnostic methodologies, sample sizes, accuracy, and limitations. The review highlights the central role of histopathology in the definitive diagnosis of GM, supported by adjunctive tools such as contrast-enhanced ultrasound, magnetic resonance imaging, and polymerase chain reaction. Imaging modalities, while valuable, often lack specificity and require histological confirmation. Emerging techniques, including quantitative imaging parameters and molecular diagnostics, offer promise in enhancing diagnostic precision. A multidisciplinary approach integrating clinical, radiological, and histopathological findings remains critical. GM diagnosis requires a nuanced approach combining traditional and emerging techniques. Histopathology remains the gold standard, but advancements in imaging and molecular diagnostics provide new avenues for improving accuracy and guiding management. The findings emphasize the need for further research and standardized diagnostic protocols to address the complexities of GM.

## Introduction and background

Granulomatous mastitis (GM) is a chronic inflammatory condition of the breast and was first reported by Kessler and Wolloch in 1972. Despite being benign, GM presents significant diagnostic challenges due to its clinical, radiological, and histopathological resemblance to malignancies and infectious breast diseases [1]. The condition primarily affects women of childbearing age, typically presenting as a unilateral, firm breast mass that may be accompanied by erythema, abscess formation, or fistulae [1]. While GM is considered idiopathic in some cases, an array of potential etiologies has been proposed, including infectious agents, autoimmune mechanisms, hormonal imbalances, and trauma [2]. The variable and nonspecific presentation of GM often results in delays in diagnosis and mismanagement, with patients frequently undergoing unnecessary surgical interventions or prolonged antibiotic therapy without definitive benefits.

One of the most significant challenges in diagnosing GM lies in distinguishing it from breast cancer. The overlapping clinical and radiological features of GM and malignancy necessitate invasive diagnostic approaches such as biopsy to rule out carcinoma [3]. Even histopathological findings, which typically reveal non-caseating granulomas, may not be conclusive without additional microbiological and serological investigations. In regions where tuberculosis is endemic, differentiating GM from tuberculous mastitis further complicates the diagnostic process [4].

The pathophysiology of GM remains poorly understood, with idiopathic cases forming the majority of diagnoses. However, emerging evidence suggests a multifactorial etiology. Infectious causes, including Corynebacterium species, have been implicated in some studies, while others propose an autoimmune mechanism based on the association of GM with systemic inflammatory conditions such as sarcoidosis and lupus [5]. Hormonal factors, particularly those related to pregnancy and lactation, are also thought to play a role, given the predilection of GM for women in reproductive age groups [6].

Radiological assessment, a cornerstone of breast disease evaluation, provides limited specificity in GM. Mammography often reveals nonspecific findings, such as asymmetric densities or irregular masses, which mimic malignancy [7]. Ultrasound, being operator-dependent, while helpful in identifying abscesses or fistulae, cannot reliably distinguish GM from other inflammatory or neoplastic conditions. MRI offers superior soft tissue characterization and may help in differentiating GM from malignancies, but its high cost and limited availability restrict its routine use [8].

Histopathology remains the diagnostic gold standard for GM, with findings of lobulocentric non-caseating granulomas without evidence of malignancy or necrosis being characteristic. However, the interpretation of biopsy samples can be challenging, as granulomatous inflammation is not specific to GM and may also be seen in infectious, foreign bodies or systemic inflammatory conditions [9]. Microbiological studies, including cultures and polymerase chain reaction (PCR), are essential for ruling out infectious causes but often yield negative results in idiopathic cases [10].

Management of GM is equally complex and underscores the importance of accurate diagnosis. The therapeutic approach varies depending on the suspected etiology, ranging from antibiotics for infectious cases to corticosteroids or immunosuppressive agents for idiopathic or autoimmune forms [11]. Surgical interventions, including wide local excision or mastectomy, are generally reserved for refractory cases or when malignancy cannot be excluded [12]. Misdiagnosis can lead to inappropriate treatments, such as unnecessary mastectomy or prolonged antibiotic courses, highlighting the need for heightened awareness and a multidisciplinary approach to GM diagnosis and management [13].

Recent advancements in imaging and molecular diagnostics hold promise for improving the diagnostic accuracy of GM. Techniques such as elastography, which evaluates tissue stiffness, and the use of AI in analyzing imaging patterns may offer new avenues for differentiation between GM and other breast pathologies [14]. Furthermore, molecular diagnostics, including next-generation sequencing and advanced PCR-based assays, have the potential to identify infectious agents or genetic markers associated with GM, aiding in its classification and management [15].

This review aims to provide a comprehensive analysis of the diagnostic challenges in GM, with a focus on current diagnostic modalities, their limitations, and emerging approaches.

## Review

The data for this review were sourced from EBSCOhost's Evidence-Based Database Service (EDS) using a comprehensive search strategy designed to identify studies relevant to the diagnostic challenges in GM. The databases included Medline, Medline Complete, CINAHL, and Web of Science (WOS). The search included terms such as "granulomatous mastitis", "diagnosis", "imaging", "biopsy", and "microbiology." Boolean operators (AND, OR) were employed to refine the results, and filters were applied to limit the selection to peer-reviewed journal articles published in English. The search spanned from the beginning to October 2024 to ensure the inclusion of contemporary studies.

Inclusion criteria for this review were studies that investigated diagnostic modalities in GM, including imaging techniques, histopathology, microbiology, and emerging diagnostic tools. Studies focusing on comparative analyses between GM and other breast diseases were prioritized. Original research articles, systematic reviews, and meta-analyses were included to provide a comprehensive perspective. Exclusion criteria were single-case reports, non-peer-reviewed articles, studies not published in English, and those that did not primarily address diagnostic methods. Studies included in the introduction were not reused to ensure broader coverage of the topic.

A total of 1261 articles were initially identified through the database search. Following the removal of duplicates and a review of abstracts for relevance, 73 studies were shortlisted for full-text evaluation. Of these, 31 met the inclusion criteria and were included in this review. The selected studies provided insights into various diagnostic techniques and their effectiveness in addressing the challenges associated with GM diagnosis. The data extracted from these studies included methodologies, sample sizes, diagnostic accuracy, and limitations of the techniques employed.

**Figure 1:**
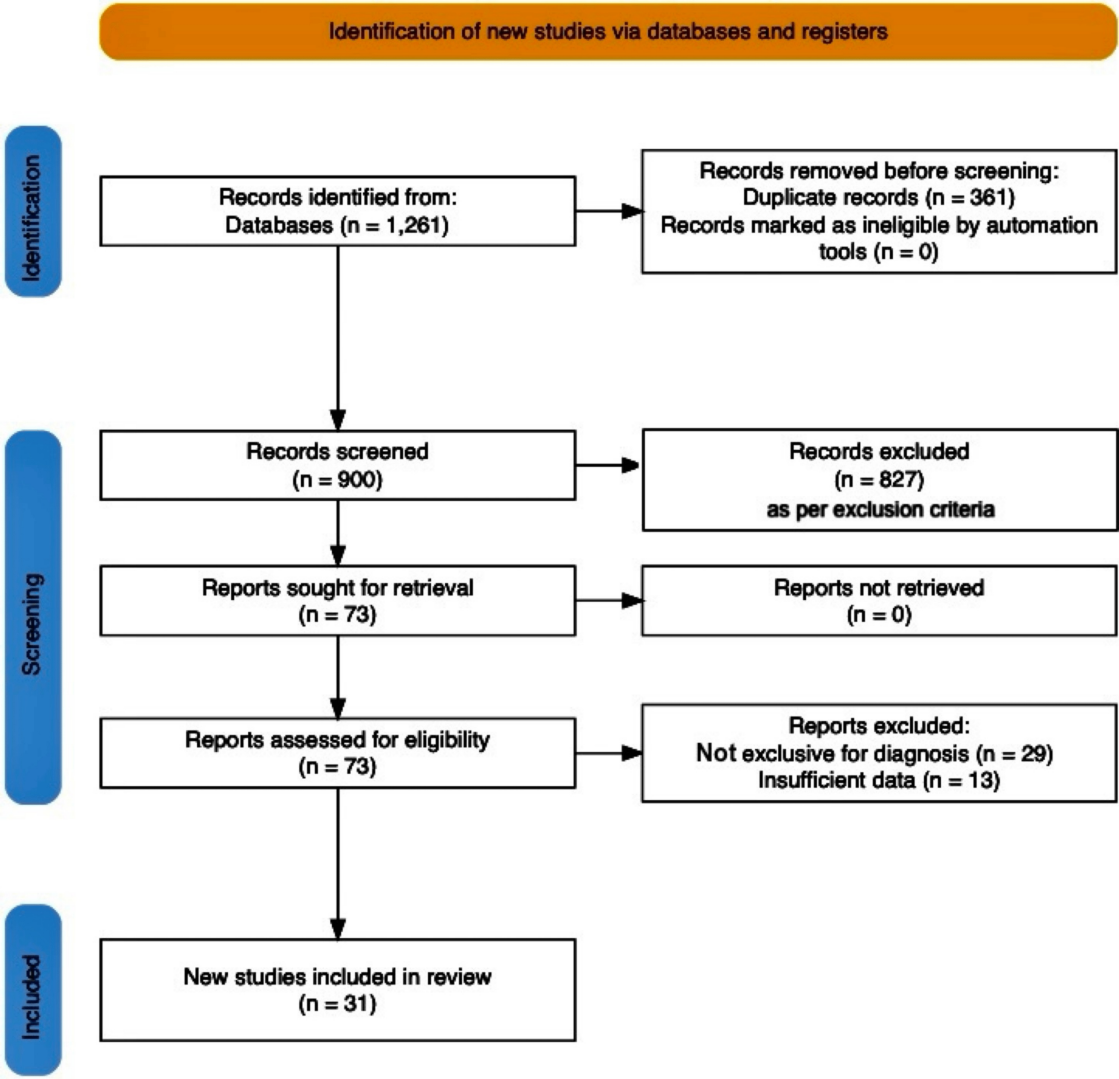
PRISMA flowchart illustrating the identification, screening, and inclusion of studies PRISMA: Preferred Reporting Items for Systematic Reviews and Meta-Analyses Identification: Represents the initial pool of records sourced from databases and other platforms, followed by duplicate removal and automated or manual exclusions. Screening: Describes the process of abstract and title screening to exclude irrelevant studies. Eligibility: Focuses on full-text articles assessed for eligibility, including reports retrieved and excluded. Inclusion: Reflects the final studies included in the narrative review for analysis and synthesis.

The review adheres to the Preferred Reporting Items for Systematic Reviews and Meta-Analyses (PRISMA) guidelines. A PRISMA flow diagram detailing the selection process is included to ensure transparency and reproducibility (Figure 1).

Additionally, a summary table listing the included studies, along with their key findings, is presented to facilitate a comprehensive understanding of the diagnostic landscape of GM (Table 1).

**Table 1:** GM diagnosis overview This table summarizes the key studies on granulomatous mastitis, presenting details such as the year of publication, author(s), study location, study title, number of patients included, study type, main findings, and conclusions. The table is organized chronologically and sorted by the reference citation of publication as required by the journal. However, the discussion in the paper is presented thematically, grouping studies by relevant topics and findings to provide a more cohesive and analytical narrative. This approach ensures a comprehensive understanding while maintaining the logical flow of the review. BC: Breast Cancer, CEUS: Contrast-Enhanced Ultrasound, DNA: Deoxyribonucleic Acid, FNAC: Fine Needle Aspiration Cytology, GLM: Granulomatous Lobular Mastitis, GM: Granulomatous Mastitis, IGM: Idiopathic Granulomatous Mastitis, MMG: Mammography, MRI: Magnetic Resonance Imaging, PCR: Polymerase Chain Reaction, TCM: Traditional Chinese Medicine, TM: Tuberculous Mastitis.

Year	Author	Place	Title	Number of Patients	Type of Study	Main Result	Conclusion
2017	Mahmodlou et al. [16]	Iran	Idiopathic granulomatous mastitis: dilemmas in diagnosis and treatment	48	Historical cohort	Histopathology fundamental; steroid therapy effective	Histopathology is crucial for accurate diagnosis; steroid therapy reduces inflammation.
2023	Esmaeil et al. [17]	Iraq	Clinical, microbiological, immunological and hormonal profiles of patients with GM	63	Cross-sectional	Bacterial species isolated; hormonal and inflammatory markers elevated	Sensitivity to trimethoprim-sulfamethoxazole supports targeted treatment.
2014	Lehman et al. [18]	USA	Imaging management of palpable breast abnormalities	-	Best practices/review	Ultrasound effective for evaluation; limited role for MRI	Ultrasound is the primary imaging tool for younger patients with palpable masses.
2007	Ozturk et al. [19]	Turkey	Granulomatous mastitis: radiological findings	9	Retrospective analysis	Wide spectrum of imaging findings; inconclusive for malignancy	Imaging findings alone are insufficient to differentiate GM from malignancy.
2013	Gautier et al. [20]	France	Chronic granulomatous mastitis: imaging, pathology and management	11	Retrospective review	Imaging features overlap with malignancies	A multidisciplinary approach is essential for accurate diagnosis.
2011	Al-Khawari et al. [21]	Kuwait	Radiologic features of granulomatous mastitis	10	Retrospective study	MRI findings often mimic malignancy	Histological confirmation is critical due to overlapping imaging features.
2010	Patel et al. [22]	USA	Idiopathic granulomatous mastitis: case reports and review of literature	2	Case reports and review	Two cases required different treatments; one responded to steroids, the other to mastectomy	Individualized treatment based on clinical presentation is crucial.
2015	Yildiz et al. [23]	Turkey	Radiologic findings of idiopathic granulomatous mastitis	30	Retrospective analysis	Irregular hypoechoic masses and collections with tubular connections suggestive of GM	Imaging findings support diagnosis but require histological confirmation.
2006	Akcan et al. [24]	Turkey	Granulomatous lobular mastitis: a complex diagnostic and therapeutic problem	21	Retrospective review	Common symptoms include breast mass and pain; imaging findings mimic cancer	Wide excision provides both therapeutic and diagnostic benefits.
2020	Yin et al. [25]	China	Differentiation between GM and breast cancer using quantitative parameters on CEUS	30	Quantitative analysis	CEUS parameters differentiated GM from cancer with high sensitivity and specificity	CEUS is a valuable tool for distinguishing GM from breast cancer.
2017	Freeman et al. [26]	USA	Idiopathic granulomatous mastitis: a diagnostic and therapeutic challenge	14	Retrospective chart review	Multimodal diagnostic approaches reduced unnecessary surgeries	Integrating imaging, histopathology, and microbiology improves diagnostic accuracy.
2022	Yin et al. [27]	China	Multimodal ultrasound assessment of mass and non-mass enhancements by MRI in GM and BC	88	Retrospective analysis	High diagnostic accuracy of ultrasound for mass lesions; MRI superior for non-mass lesions	Combined imaging modalities improve diagnostic accuracy in complex cases.
2010	Ocal et al. [28]	Turkey	Granulomatous mastitis: clinical, pathological features, and management	16	Clinical study	Wide excision with negative margins effective; steroids used in resistant cases	Surgery combined with steroids reduces recurrence in resistant cases.
2014	Kiyak et al. [29]	Turkey	Management of idiopathic granulomatous mastitis: dilemmas in diagnosis and treatment	24	Retrospective review	Common symptoms included painful, firm, ill-defined masses. US showed parenchymal heterogeneity and abscesses; MMG revealed increased asymmetric density. Wide local excision was performed in 62.5% of patients	Histopathologic confirmation is mandatory for accurate diagnosis. US findings suggest inflammatory processes but cannot exclude malignancy.
2016	Fazzio et al. [30]	USA	Idiopathic granulomatous mastitis: imaging update and review	17	Retrospective review	Imaging findings mimic malignancy; MRI valuable for assessing extent and response to therapy	Awareness of imaging findings prevents misdiagnosis and overtreatment.
2019	Martinez-Ramos et al. [31]	Spain	Idiopathic granulomatous mastitis: a systematic review of 3060 patients	3060	Systematic review	No consensus on optimal treatment; high recurrence rates with medical therapy alone	Surgical and medical therapies combined improve long-term outcomes.
2024	Dilaveri et al. [32]	USA	Idiopathic granulomatous mastitis: a narrative review	-	Review	Medical management preferred; surgery reserved for refractory cases	Observation, steroids, and immunosuppressants effective in most cases.
2018	Pluguez-Turull et al. [33]	USA	Idiopathic granulomatous mastitis: manifestations at multimodality imaging and pitfalls	-	Review	Imaging findings mimic malignancy; multidisciplinary approach necessary for diagnosis	Early imaging combined with biopsy prevents misdiagnosis and overtreatment.
2004	Tse GM et al. [34]	China	Granulomatous mastitis: a clinicopathological review of 26 cases	26	Retrospective Review	Granulomas present in 100% of cases; lymphocytic lobulitis in 68% and necrosis in 11%; special stains and cultures negative for micro-organisms; one case showed Mycobacterium DNA via PCR	PCR aids in identifying infectious etiologies and guiding treatment.
2020	Alikhassi et al. [35]	Iran	Imaging features of granulomatous mastitis in 36 patients with new sonographic signs	36	Prospective study	Sonographic findings include hypoechoic masses with duct ectasia and pseudocyst appearance	Sonography highly suggests GM in appropriate clinical context.
2016	Yilmaz et al. [36]	Turkey	Magnetic resonance imaging features of idiopathic granulomatous mastitis	37	Retrospective analysis	MRI findings suggestive but nonspecific	MRI aids in delineating disease extent but requires histopathological confirmation.
2019	Hasbahceci et al. [37]	Turkey	Use of Imaging for the diagnosis of idiopathic granulomatous mastitis: a clinician’s perspective	948	Review	Common imaging findings include tubular hypoechoic masses with parenchymal distortion	Imaging findings guide clinical decisions but require histological confirmation.
2022	Velidedeoglu et al. [38]	Turkey	Idiopathic granulomatous mastitis: introducing a diagnostic algorithm	152	Retrospective cohort	High recurrence rates with medical management; proposed diagnostic and treatment algorithm	Multidisciplinary diagnostic strategies reduce recurrence and improve management.
2016	Poyraz et al. [39]	Turkey	Magnetic resonance imaging features of idiopathic granulomatous mastitis	20	Retrospective analysis	MRI shows rim enhancement and non-mass lesions; nonspecific findings	MRI aids in assessing extent and response to therapy but cannot replace biopsy.
2006	Lee et al. [40]	Korea	Radiologic and clinical features of idiopathic granulomatous lobular mastitis	12	Retrospective analysis	Sonographic features include irregular hypoechoic masses with minimal parenchymal distortion	Imaging findings aid in differentiation, but histology is necessary for definitive diagnosis.
2008	Al-Khaffaf et al. [41]	UK	Idiopathic granulomatous mastitis: a 25-year experience	18	Retrospective review	Younger patients with recent childbirth were most affected; recurrence noted	Diagnosis requires high clinical suspicion; management should be supportive.
2016	Seo et al. [42]	Korea	Differential diagnosis in idiopathic granulomatous mastitis and tuberculous mastitis	68	Retrospective analysis	TM associated with axillary lymphadenopathy and cancer-mimicking findings	Accurate differentiation between IGM and TM improves therapeutic outcomes.
2016	Helal et al. [43]	Egypt	Idiopathic granulomatous mastitis: cytologic and histologic study of 65 Egyptian patients	65	Cytologic and histologic	FNAC has high accuracy in excluding malignancy; bacterial presence noted in cystic vacuoles	FNAC can assist in diagnosis, but histology remains essential for definitive diagnosis.
2024	Ning et al. [44]	China	Imaging manifestations and treatment efficacy of Granulomatous Lobular Mastitis with TCM	29	Retrospective analysis	MRI changes correlated with clinical improvement post-TCM therapy	Combining internal and external TCM therapy improves outcomes in GLM patients.
2018	Wolfrum et al. [45]	Germany	Granulomatous mastitis: a therapeutic and diagnostic challenge	-	Review	Imaging mimics cancer; medical therapy followed by surgery in persistent cases	A registry is needed for better understanding of the disease.
2019	Maione et al. [46]	Italy	Diagnostic techniques and multidisciplinary approach in idiopathic granulomatous mastitis	-	Review	Multidisciplinary approaches avoid unnecessary surgeries	Advances in imaging and medical therapy reduce the need for invasive procedures.

Clinical features and presentation

GM presents a significant diagnostic challenge due to its nonspecific and diverse clinical manifestations. Patients frequently report painful breast lumps, erythema, and abscess formation, often mimicking conditions such as inflammatory breast carcinoma or infectious mastitis [16,17,19]. In their cohort of 48 patients, Mahmodlou et al. [16] noted that while 68.75% presented with palpable masses, only 20.83% exhibited purulent drainage, emphasizing the variability in symptoms. Esmaeil et al. [17] found that systemic symptoms such as fever and chills, along with elevated inflammatory markers such as C-reactive protein and IL-6, could further complicate differentiation from infectious etiologies.

Notably, demographic factors provide critical diagnostic clues. GM predominantly affects premenopausal women with a history of breastfeeding, as highlighted in studies by Poyraz et al. [39] and Freeman et al. [26]. This association with hormonal fluctuations and lactational changes supports hypotheses of milk stasis or autoimmunity as contributing factors. However, regional factors, such as tuberculosis prevalence, necessitate heightened clinical suspicion for alternative etiologies, particularly in endemic areas [42,43].

Histopathological diagnosis: the gold standard

Histopathology remains the definitive diagnostic modality for GM, with lobulocentric granulomas serving as the hallmark finding. Studies consistently underscore the necessity of tissue biopsy, as imaging alone is often inconclusive [16,41]. While FNAC offers a less invasive alternative, it lacks the sensitivity to confirm diagnosis independently, as shown by Helal et al. [43]. Core needle biopsy remains the preferred approach for obtaining adequate tissue samples for histological evaluation.

However, histopathological overlap with infectious or systemic granulomatous conditions poses significant challenges. Seo et al. [42] reported that 10% of cases initially diagnosed as idiopathic GM were later confirmed as tuberculous mastitis following microbiological testing. This highlights the importance of integrating histopathological findings with microbiological and clinical data to ensure diagnostic accuracy.

Imaging modalities: strengths and weaknesses

Imaging plays an essential adjunctive role in diagnosing GM, helping to delineate disease extent and differentiate it from malignancies. Mammography and ultrasound are the primary imaging modalities used, yet their findings are often nonspecific. Ozturk et al. [19] reported that mammographic features such as asymmetric densities and spiculated masses are common but indistinguishable from malignancies. Similarly, hypoechoic masses with irregular margins are typical ultrasound findings, as noted by Poyraz et al. [39]. These imaging features, while suggestive, necessitate histological confirmation to avoid misdiagnosis.

Advanced imaging techniques offer additional diagnostic insights. Yilmaz et al. [36] demonstrated that MRI, particularly dynamic contrast-enhanced MRI, provides valuable information about lesion morphology, such as rim enhancement and clustered-ring non-mass lesions. These findings are particularly useful in identifying abscesses and ductal ectasia, which are common in GM. Moreover, dynamic contrast patterns can help differentiate GM from malignancies, as highlighted by Yin et al. [25]. Despite these advantages, the high cost and limited availability of MRI restrict its widespread use, especially in resource-constrained settings.

Emerging modalities such as elastography and contrast-enhanced ultrasound (CEUS) show promise in improving diagnostic precision. Yin et al. [25] reported that CEUS demonstrated high sensitivity and specificity in distinguishing GM from breast cancer based on quantitative parameters such as time-to-peak and wash-in slope. While these technologies hold potential, further validation in larger cohorts is necessary to establish their clinical utility.

Molecular and microbiological tools

The incorporation of molecular diagnostics, such as PCR, into the diagnostic workflow has significantly enhanced the identification of infectious causes of GM. Tse et al. [34] demonstrated that PCR aids in identifying infectious etiologies and guiding treatment. Similarly, Esmaeil et al. [17] highlighted the value of bacterial cultures in identifying nine distinct bacterial species, underscoring the role of microbiological evaluation in guiding therapy.

However, idiopathic GM remains a diagnosis of exclusion, with negative culture results in a significant proportion of cases [43]. This underscores the importance of a multidisciplinary approach, combining clinical, histopathological, and molecular findings to achieve an accurate diagnosis.

Comparison with differential diagnoses

Differentiating GM from other granulomatous and malignant conditions remains a critical challenge. Tuberculous mastitis, for instance, shares clinical and radiological features with GM, necessitating microbiological testing for definitive diagnosis. Seo et al. [42] emphasized the role of axillary lymphadenopathy and positive culture results in distinguishing tuberculosis from idiopathic GM. Similarly, inflammatory breast carcinoma often mimics GM, both clinically and radiologically, as noted by Al-Khawari et al. [21]. In such cases, targeted imaging findings, such as rim enhancement and ductal ectasia, provide valuable diagnostic clues.

Misdiagnosis can lead to unnecessary interventions, including mastectomy or inappropriate antibiotic therapy. Martinez-Ramos et al. [31] highlighted that accurate differentiation between GM and its mimics significantly reduced the rate of invasive procedures, emphasizing the importance of a comprehensive diagnostic strategy.

Combined approaches and challenges

The integration of histopathology, imaging, and molecular diagnostics offers the most robust approach to diagnosing GM. Freeman et al. [26] demonstrated that combining imaging with PCR increased diagnostic confidence and reduced unnecessary surgeries. Similarly, Gautier et al. [20] highlighted the synergy between imaging and histopathology in achieving a more accurate diagnosis.

However, implementing multimodal approaches poses logistical and financial challenges, particularly in resource-limited settings. Studies [17,29,31] emphasize the need for standardized diagnostic protocols to streamline the diagnostic process and reduce variability in clinical practice.

Treatment implications based on diagnostic accuracy

Accurate diagnosis directly impacts treatment outcomes in GM. Medical management, including corticosteroids and immunosuppressants, remains the mainstay of therapy, with surgery reserved for refractory or recurrent cases [31,38]. Misdiagnosis, however, often leads to overtreatment, such as unnecessary mastectomies or prolonged antibiotic courses. Freeman et al. [26] reported that precise diagnosis reduced the rate of surgical interventions by 30%, highlighting the clinical and economic benefits of accurate diagnostic practices.

Advanced imaging modalities not only aid in diagnosis but also guide treatment planning. Yilmaz et al. [36] noted that MRI findings could delineate disease extent and monitor therapeutic response, thereby informing clinical decision-making.

Interpretation of bias 

The studies reviewed in this narrative systematically highlight a range of potential biases that impact their findings’ generalizability and reliability. While most studies demonstrated low bias in the classification of exposures and reporting outcomes, moderate bias was frequently noted in the areas of confounding and selection. This is largely due to the retrospective nature of many studies and the inherent variability in patient populations. For instance, studies such as Kiyak et al. [29] and Yin et al. [25] showed moderate risk in confounding due to heterogeneous treatment protocols and diagnostic approaches, reflecting the lack of standardized guidelines for managing idiopathic granulomatous mastitis (IGM). Similarly, selection bias was moderate in several studies, as patient samples were often drawn from single centers with limited demographic diversity, potentially restricting external validity. Despite these limitations, the overall risk was generally acceptable, with most studies achieving low bias in critical domains such as outcome measurement and reporting. This indicates that while findings should be interpreted with caution, they provide valuable insights into IGM diagnostics and management when considered collectively (Table 2).

**Table 2:** Risk of bias table using ROBINS-I tool ROBINS-I: Risk of Bias in Non-randomized Studies of Interventions How to interpret the table: C (Confounding): Studies may have uncontrolled confounders influencing outcomes (e.g., patient demographics, comorbidities). SB (Selection Bias): Studies with retrospective designs or poorly defined inclusion/exclusion criteria may have moderate bias. CE (Classification of Exposures): Consistently low bias if exposures are well-defined and measured accurately. I (Deviations From Interventions): Moderate bias if there are differences in how interventions were applied across participants. mD (Missing Data): Low bias if data are complete or imputation is appropriately used. O (Outcome Measurement): Moderate bias if blinding is not used or measurement techniques are inconsistent. R (Reporting Bias): Moderate bias if selective reporting of outcomes is suspected. OR (Overall Risk): Each domain is evaluated with traffic light coding: Green (Low Risk), Yellow (Moderate Risk), Red (High Risk).

Study	C	SB	THIS	I	mD	THE	R	OR
Mahmodlou et al. [16]	🟡	🟡	🟢	🟢	🟢	🟡	🟡	🟡
Esmaeil et al. [17]	🟡	🟢	🟢	🟡	🟡	🟡	🟡	🟡
Lehman et al. [18]	🟢	🟢	🟢	🟡	🟡	🟢	🟢	🟡
Ozturk et al. [19]	🟡	🟡	🟢	🟢	🟡	🟢	🟡	🟡
Gautier et al. [20]	🟡	🟢	🟢	🟢	🟡	🟢	🟡	🟡
Al-Khawari et al. [21]	🟡	🟡	🟢	🟢	🟢	🟡	🟢	🟡
Patel et al. [22]	🟡	🟡	🟢	🟡	🟡	🟡	🟡	🟡
Yildiz et al. [23]	🟢	🟡	🟢	🟡	🟡	🟡	🟢	🟡
Akcan et al. [24]	🟡	🟢	🟢	🟢	🟢	🟢	🟢	🟢
Yin et al. [25]	🟡	🟡	🟢	🟡	🟢	🟡	🟡	🟡
Freeman et al. [26]	🟡	🟡	🟢	🟡	🟡	🟡	🟡	🟡
Yin et al. [27]	🟡	🟡	🟢	🟡	🟢	🟡	🟡	🟡
Ocal et al. [28]	🟢	🟡	🟢	🟢	🟢	🟢	🟢	🟢
Kiyak et al. [29]	🟡	🟡	🟢	🟡	🟡	🟡	🟢	🟡
Fazzio et al. [30]	🟡	🟡	🟢	🟡	🟢	🟡	🟡	🟡
Martinez-Ramos et al. [31]	🟡	🟢	🟢	🟢	🟡	🟢	🟡	🟡
Dilaveri et al. [32]	🟡	🟡	🟢	🟢	🟢	🟢	🟡	🟡
Pluguez-Turull et al. [33]	🟡	🟢	🟢	🟡	🟢	🟡	🟡	🟡
Tse et al. [34]	🟢	🟢	🟢	🟡	🟢	🟢	🟢	🟢
Alikhassi et al. [35]	🟢	🟢	🟢	🟡	🟢	🟢	🟡	🟢
Yilmaz et al. [36]	🟢	🟡	🟢	🟡	🟡	🟢	🟢	🟡
Hasbahceci et al. [37]	🟡	🟡	🟢	🟢	🟡	🟡	🟡	🟡
Velidedeoglu et al. [38]	🟡	🟡	🟢	🟢	🟢	🟢	🟡	🟡
Poyraz et al. [39]	🟡	🟢	🟢	🟢	🟡	🟡	🟢	🟡
Lee et al. [40]	🟡	🟢	🟢	🟢	🟢	🟢	🟡	🟡
Al-Khaffaf et al. [41]	🟢	🟢	🟢	🟢	🟢	🟢	🟢	🟢
Seo et al. [42]	🟢	🟡	🟢	🟡	🟢	🟢	🟢	🟢
Helal et al. [43]	🟡	🟡	🟢	🟢	🟡	🟡	🟢	🟡
Ning et al. [44]	🟢	🟡	🟢	🟡	🟢	🟢	🟢	🟡
Wolfrum et al. [45]	🟢	🟢	🟢	🟢	🟢	🟡	🟢	🟢
Maione et al. [46]	🟢	🟢	🟢	🟡	🟢	🟢	🟢	🟢

Summary

The summary table provides a detailed overview of the strengths and weaknesses of various diagnostic modalities based on the reviewed studies. This summary highlights the utility and limitations of each approach, offering a comparative perspective to guide clinicians in selecting the most appropriate tools for accurate diagnosis and effective management of GM. Clinicians must note that the summary table is based on the review and provided only as a guide (Table 3).

**Table 3:** Strengths and weaknesses of diagnostic modalities for GM The table summarizes the strengths and weaknesses of various diagnostic modalities for GM, including histopathology, imaging techniques, and molecular diagnostics. CEUS: Contrast-Enhanced Ultrasound, FNAC: Fine Needle Aspiration Cytology, GM: Granulomatous Mastitis, MRI: Magnetic Resonance Imaging, PCR: Polymerase Chain Reaction, US: Ultrasound. These terms are integral to understanding the diagnostic approaches discussed and are based on evidence synthesized from the cited studies.

Diagnostic Modality	Strengths	Weaknesses
Histopathology [16,18,24,30]	Gold standard; provides definitive diagnosis; identifies granulomas	Invasive procedure; requires expertise for interpretation; potential sampling errors
US [19,23,35]	Widely available; identifies hypoechoic masses and abscesses; low cost	Nonspecific findings; cannot differentiate GM from malignancies without histological confirmation
CEUS [27,44]	Enhances vascular details; aids in distinguishing benign vs. malignant lesions	Limited availability; requires specialized training; still lacks standalone diagnostic specificity
MRI [19,30,36,39]	High sensitivity; useful for extent assessment; identifies rim-enhancing lesions	Expensive; nonspecific findings; cannot replace histopathology for definitive diagnosis
PCR [43]	Detects specific pathogens (e.g., Corynebacterium); useful for infectious GM	Limited application; requires advanced lab facilities; negative result does not exclude GM
FNAC [18,43]	Minimally invasive; excludes malignancy effectively	Cannot provide conclusive diagnosis of GM; potential sampling errors
Mammography [19,23,28]	Identifies asymmetrical densities and structural distortions	Poor sensitivity in young, dense breasts; findings overlap significantly with malignancies
Quantitative imaging techniques [27,30,36]	Promising in providing objective metrics (e.g., time-to-peak, vascularity)	Limited validation; requires further research for routine clinical use

Knowledge gaps and future directions

Despite advances in diagnostic modalities, significant gaps remain in understanding and managing GM. The lack of standardized diagnostic criteria and the variability in imaging and histopathological findings contribute to diagnostic uncertainty. Moreover, the etiology of idiopathic GM remains unclear, with hormonal, autoimmune, and infectious factors implicated [16,31,43].

Future research should focus on developing diagnostic algorithms that integrate clinical, imaging, and molecular data. Multicenter studies are needed to evaluate the efficacy of emerging diagnostic modalities and their impact on patient outcomes. Establishing international registries for GM could provide valuable insights into its epidemiology, etiology, and optimal management strategies.

## Conclusions

GM remains a challenging entity to diagnose and manage due to its overlapping clinical and imaging features with malignant and infectious breast conditions. Histopathology continues to be the cornerstone of definitive diagnosis, with emerging techniques such as PCR providing additional precision in identifying infectious etiologies. Imaging modalities, including ultrasound, CEUS, and MRI, play crucial roles in initial evaluation and disease monitoring but lack specificity for standalone diagnosis. The literature review highlights the importance of a multidisciplinary approach, combining clinical, radiological, and histopathological inputs to improve diagnostic accuracy and avoid unnecessary interventions. As the understanding of GM continues to evolve, integrating newer diagnostic tools and individualized treatment protocols will be key to optimizing patient outcomes.
